# Systematic review and meta‐analysis of implant‐specific outcomes in inlay and onlay patellofemoral arthroplasty for isolated patellofemoral osteoarthritis

**DOI:** 10.1002/jeo2.70541

**Published:** 2025-11-14

**Authors:** Pietro Cimatti, Martina Rocchi, Benedetta Dallari, Marco Corzani, Silvio Caravelli, Massimiliano Mosca, Luca Macchiarola, Mattia Morri, Giulio Maria Marcheggiani Muccioli, Stefano Zaffagnini, Dante Dallari

**Affiliations:** ^1^ Reconstructive Orthopaedic Surgery Innovative Techniques–Musculoskeletal Tissue Bank IRCCS Istituto Ortopedico Rizzoli Bologna Italy; ^2^ Bentivoglio Orthopaedic Unit IRCCS Istituto Ortopedico Rizzoli Bologna Italy; ^3^ Department of Orthopedics and Trauma Surgery Fondazione Casa Sollievo Della Sofferenza IRCCS San Giovanni Rotondo Italy; ^4^ Nursing, Technical and Rehabilitation Assistance Service IRCCS Istituto Ortopedico Rizzoli Bologna Italy; ^5^ 2nd Orthopedic and Traumatologic Clinic IRCCS Istituto Ortopedico Rizzoli Bologna Italy; ^6^ Dipartimento di Scienze Biomediche e Neuromotorie DIBINEM University of Bologna Bologna Italy

**Keywords:** femoropatellar replacement, patellofemoral arthroplasty, patellofemoral joint, patellofemoral osteoarthritis

## Abstract

**Purpose:**

The purpose of this systematic review was to compare clinical outcomes and complication rates between inlay and onlay patellofemoral arthroplasty (PFA) for isolated patellofemoral osteoarthritis (PFOA). A secondary objective was to evaluate implant‐specific performance among various prosthetic designs.

**Methods:**

A comprehensive literature search was conducted using PubMed/MEDLINE, EMBASE and the Cochrane Database to identify studies published between January 1980 and 20 January 2025. Studies were included if they reported functional outcomes and complication rates for inlay or onlay PFA with a mean follow‐up of 5 years. The majority of included studies were Level III or IV observational studies; therefore, the strength of the evidence is limited and conclusions should be interpreted with caution.

**Results:**

Seventy‐six studies met the inclusion criteria, encompassing 4484 patients and 5084 implants. Onlay prostheses were associated with significantly higher rates of good to excellent outcomes (88% vs. 76%, *p* < 0.001), lower complication rates (7% vs. 19%, *p* < 0.001), and lower revision rates (4% vs. 8% TKA conversions, *p* = 0.02) compared to inlay implants. Among onlay designs, the Avon (Stryker) and Gender Solutions (Zimmer) prostheses showed the best results, with 90% and 87% success rates, respectively. The Lubinus (Link) inlay prosthesis demonstrated the poorest performance, with high complication and revision rates. Patellar maltracking was more frequent in the inlay group (4% vs. 1%), though not statistically significant. Infection and aseptic loosening rates were negligible in both groups.

**Conclusion:**

Onlay PFA appears to offer clinical advantages over inlay designs in the treatment of isolated PFOA, with trends toward superior functional outcomes, lower complication and revision rates, and more consistent performance across models. However, these results should be interpreted in light of the predominance of non‐randomized studies and the heterogeneity of the available literature. The findings support the preferential use of onlay implants, particularly the Avon and Gender Solutions designs. Implant selection should be guided by evidence‐based performance and patient‐specific anatomical considerations.

**Level of Evidence:**

Level IV.

**Abbreviations**
iKA: inverse kinematic alignment
PFA: patellofemoral arthroplasty
PFOA: patellofemoral osteoarthritis
PQT: proximal quadriceps tendon
TKA: total knee arthroplasty

## INTRODUCTION

Patellofemoral osteoarthritis (PFOA) is a common and often disabling condition that primarily affects middle‐aged and elderly individuals. Radiographic evidence of PFOA is present in approximately 10%–24% of symptomatic knees, particularly in women over the age of 55 [1, 10, 11].

Although the disease can be secondary to trauma, instability, or dysplasia, a substantial proportion of cases remain idiopathic [52, 61]. The pathogenesis of PFOA is multifactorial, involving factors such as malalignment, trochlear dysplasia, altered biomechanics and soft tissue imbalance, with many patients exhibiting isolated involvement of the patellofemoral compartment [27, 29, 35]. Despite its high prevalence, PFOA is frequently underdiagnosed and undertreated, especially in its early stages. Conservative management—including weight loss, physiotherapy, bracing, taping, anti‐inflammatory medications and activity modification—is generally considered the first‐line treatment [9]. When nonoperative approaches fail, surgical options such as lateral release, tibial tubercle transfer, cartilage restoration or arthroscopy may be considered, although their long‐term efficacy remains variable [22, 45, 60].

In more advanced cases, surgical intervention often involves joint resurfacing. While total knee arthroplasty (TKA) has traditionally been the standard treatment for end‐stage osteoarthritis, patellofemoral arthroplasty (PFA) has emerged as a viable alternative for patients with isolated PFOA and preserved tibiofemoral compartments. Early PFA designs were associated with high complication and failure rates, limiting their widespread adoption [8, 20, 41]. However, advances in implant design, surgical technique, and patient selection have renewed interest in PFA, particularly with second‐generation onlay implants such as the Avon and FPV, which provide improved patellar tracking and joint congruity [2, 3, 14, 47, 50].

The anatomy and mechanics of the trochlea remain pivotal in patellofemoral outcomes—both in PFA and TKA. A recent comparative study evaluating two cruciate‐sacrificing TKA systems (ROCC vs. LCS‐RP) demonstrated that deeper trochlear geometry in the ROCC system was associated with improved patellar centration and superior clinical outcomes in patellar nonresurfacing procedures, highlighting the relevance of trochlear design in stabilizing the native patella [32]. Furthermore, the position of the proximal quadriceps tendon (PQT) relative to the prosthetic trochlea has been proposed as a potential predictor of outcomes following kinematically aligned TKA. However, a large cohort study found no significant association between lateral PQT positioning and patient‐reported outcomes or patellofemoral instability at two years postoperatively, suggesting that this parameter may not be clinically relevant in all settings [50]. In addition, the restoration of native patellofemoral joint line through patient‐specific alignment strategies—such as inverse kinematic alignment (iKA)—has gained attention for its potential to enhance patellofemoral tracking and patient satisfaction, even when using conventional instrumentation [52]. PFA offers several advantages over TKA in appropriately selected patients, including preservation of native joint kinematics, less invasive surgery, faster recovery, and easier conversion to TKA if needed [18, 19, 26, 40, 42, 43, 51, 59]. Typical indications for isolated PFA include failure of conservative treatment, radiographic evidence of isolated patellofemoral degeneration, absence of tibiofemoral arthritis, intact ligaments and menisci, and normal or correctable patellar tracking [8, 13, 44]. However, outcomes remain variable, with a substantial proportion of failures attributed to progression of tibiofemoral arthritis or patellar maltracking [12, 15]. The ongoing debate between inlay and onlay prosthetic designs further complicates implant selection.

Onlay implants, such as the Avon and FPV, allow greater control over component positioning and rotational alignment, but may alter joint anatomy and increase the risk of overstuffing [13, 17, 21, 25, 28, 38, 44, 49]. In contrast, inlay designs, like the HemiCap Wave, aim to replicate native trochlear geometry by mapping the patient′s articular surface intraoperatively, potentially reducing mechanical complications and preserving soft tissue balance. Despite promising biomechanical rationale, limited data exists regarding the clinical performance of modern inlay prostheses [31, 33, 49].

Moreover, surgical approach may influence outcomes. The medial parapatellar arthrotomy, the most commonly used approach, has been associated with disruption of patellar vascularity and postoperative complications such as instability and anterior knee pain [23, 24, 36, 37, 39]. The lateral parapatellar approach may mitigate some of these risks, though it is less familiar and more technically demanding [34, 58]. Given the limited data currently available in the literature, this systematic review aimed to assess the outcomes and complication rates of inlay versus onlay PFA for isolated PFOA. Furthermore, although PFA implants are often grouped as inlay or onlay designs, clinical outcomes can vary substantially not only between these two categories but also among specific implants. This heterogeneity underscores the need for implant‐specific comparisons, which represent the focus of the present systematic review.

## METHODS

### Search strategy

This systematic review and meta‐analysis was performed in accordance with the Preferred Reporting Items for Systematic Reviews and Meta‐Analyses (PRISMA) guidelines. The focus of this study was on the research question regarding outcomes and complications rates between inlay and onlay PFA for isolated PFOA. A thorough search was carried out in PubMed/MEDLINE, EMBASE and the Cochrane Database of Systematic Reviews for articles published from January 1980 to 20th January 2025. The search parameters used were: (patell*) AND ((patellofemoral) OR (patellafemoral) OR (patellofemoral joint) OR (femoropatell*) OR (femoro‐patell*)) AND ((arthroplasty) OR (replacement) OR (resurfacing) OR (prosthesis) AND ((patellofemoral arthritis) OR (femoro‐patellar arthritis) OR (patellofemoral osteoarthritis) OR (femoro‐patellar osteoarthritis) OR (femoropatellar arthritis) OR (femoropatellar osteoarthritis)) AND ((chondral) OR (cartilage defect*) OR (ailment) OR (injury) OR (damage) OR (chondropathy)), with words occurring in the title and/or abstract of the article.

A systematic search was conducted across electronic databases using various combinations of the previously specified keywords. Two independent reviewers screened all identified studies, with discrepancies resolved through discussion or consultation with a third reviewer. The review protocol was submitted for registration in the PROSPERO database (_ID 1084259_). Data extraction was carried out by at least two reviewers using standardized forms that included predefined outcome measures.

### Eligibility criteria

Two reviewers (P.C. and M.R.) independently evaluated the titles and abstracts of the identified studies. To be considered for inclusion, studies needed to report on outcomes and complications rates between inlay and onlay PFA for isolated PFOA. The inclusion criteria required the study to be a clinical trial discussing the PFA for isolated PFOA, with no limitations based on language, publication date or follow‐up length. Both prospective and retrospective studies were permitted, and the level of evidence was classified according to the Oxford Centre for Evidence‐Based Medicine guidelines. Studies involving fewer than 10 patients, focusing solely on biomechanics, lacking radiological and functional outcomes were excluded.

### Study selection and data extraction

**Figure 1:**
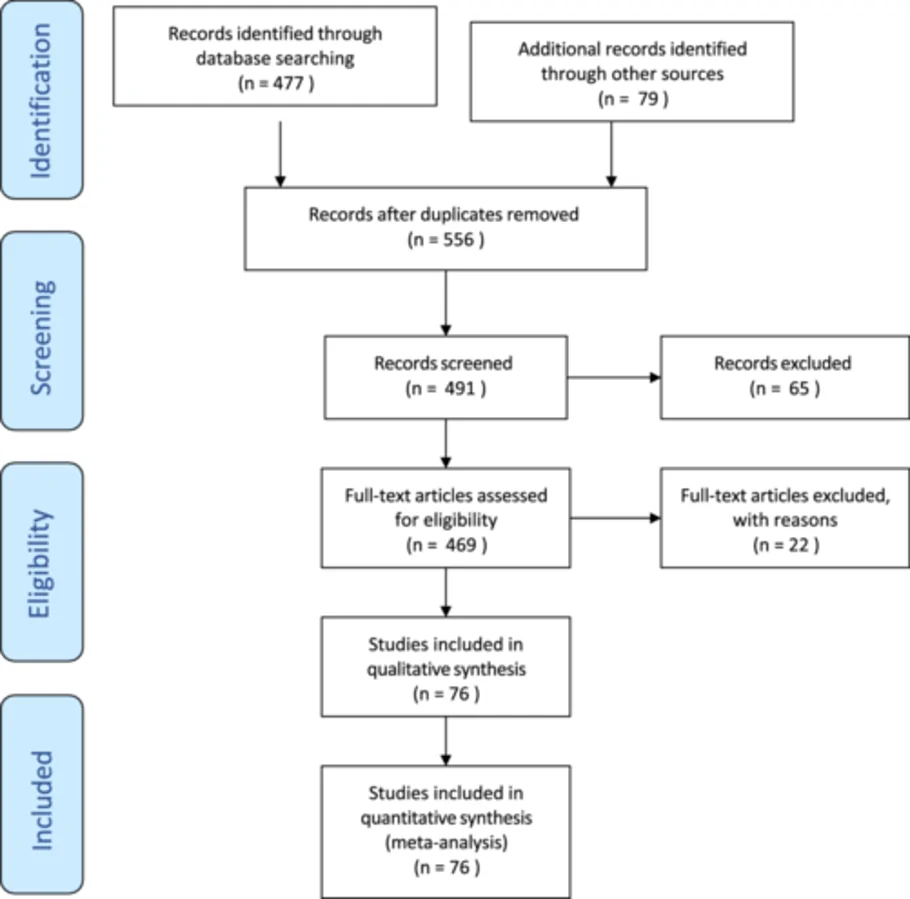
Prisma flow diagram.

The search and preliminary screening of abstracts were conducted by two independent reviewers, who resolved any discrepancies through discussion. 556 studies were initially identified. A primary screening of abstracts narrowed this to 469 studies, which were then subjected to full‐text review. Ultimately, 390 studies were excluded due to the absence of patellofemoral prosthesis implantation, leading to 76 studies included in the analysis (Figure 1). The quality of these studies was appraised using the Newcastle–Ottawa Quality Assessment Scale, with no minimum quality threshold established to promote inclusivity (Table 1).

**Table 1:** Quality assessment of the studies using the Newcastle‐Ottawa scale for cohort studies.

Study exposed dy	First author	The exposed cohort	Selection of the non expo sed coho rt	Ascertainment of exposure	Demonstration that outcome of interest was not present at start of study	Comparability of cohorts on the basis of the design or analysis	Assessment of outcome	Was follow‐up long enough for outcomes to occur	Adequacy of follow‐up of cohorts	N O S
1	Cartier	*		*	*		*	*	*	5
2	Argenso n	*		*	*		*	*	*	6
3	Utukuri	*		*	*		*	*	*	6
4	Tauro	*		*	*		*	*	*	7
5	Mercha nt	*		*	*		*	*	*	7
6	Krajca‐Radcliff e	*		*	*		*	*		5
7	Smith	*		*	*		*	*	*	6
8	Christop her E. Ackroyd	*		*	*		*	*	*	7
9	AndersOdgaar d	*			*		*	*	*	7
10	Enes Uluyardi mci	*		*	*		*	*	*	6
11	Martíne z‐ Sánche z JA	*		*	*	*	*	*	*	7
12	Jens Ole Laursen	*		*	*		*	*	*	6
13	Burger, Joost A	*		*	*		*		*	6
14	A. Odgaar d	*	*	*	*	**	*	*	*	9
15	Hernigo u P, Caton J.	*		*	*		*	*	*	6
16	Konan S, Haddad FS	*		*	*		*	*	*	6
17	Rogers JT	*		*	*		*	*	*	6
18	Patel A, Haider Z	*		*	*		*	*	*	6
19	Imhoff AB	*		*	*	**	*	*	*	9
20	Kamikov ski I	*	*	*	*	*	*	*	*	8
21	Board TN	*		*	*		*	*	*	6
22	Smith AM	*		*	*		*	*	*	6
23	Sisto DJ	*		*	*		*	*	*	6
24	Jeong	*	*	*	*	*	*	*	*	8
25	Dai Y	*		*	*	*	*	*		7
26	Valoros o	*		*	*		*	*	*	6
27	Ajnin	*		*	*		*	*	*	6
28	Van Engen	*		*	*		*	*	*	7
29	Ramos P	*		*	*		*	*		5
30	Osarum wense D	*		*	*	*	*	*	*	7
31	Mont M	*		*	*		*	*	*	6
32	PerroneFL	*	*	*	*	*	*	*		7
33	Kooijma n HJ	*		*	*		*	*		5
34	Lonner JH	*	*	*	*		*			5
35	Lonner JH	*	*	*	*		*			5
36	Mofdi A	*		*	*		*	*	*	6
37	Metcalfe AJ	*		*	*	*	*	*	*	7
38	Rammo han R	*		*	*	*	*	*		6
39	Willeken s P	*		*	*		*	*		5
40	Ahearn N	*		*	*		*	*	*	6
41	Akhbari P	*		*	*		*	*	*	6
42	Al‐ Hadithy N	*		*	*		*	*	*	6
43	Beckmann J	*		*	*	*	*	*	*	7
44	Dahm DL	*		*	*	*	*	*	*	7
45	Davies AP	*		*	*		*	*	*	6
46	Goh GS	*		*	*		*	*	*	7
47	Imhof AB	*		*	*		*	*	*	6
48	Imhof AB	*		*	*		*	*	*	6
49	Feucht MJ	*	*	*	*	*	*	*	*	8
50	Feucht MJ	*	*	*	*	**	*	*	*	9
51	Pogorze lski J	*		*	*	*	*	*	*	7
52	Willekens	*		*	*	*	*	*	*	7
53	Beitzel K	*		*	*		*	*	*	6
54	Bernard CD	*	*	*	*	*	*	*	*	8
55	Arnbjors son AH	*		*	*		*	*	*	6
56	Bohu Y	*		*	*	*	*	*	*	7
57	Cartier P	*		*	*		*	*	*	6
58	Cartier P	*		*	*			*		4
59	Hoogerv orst P	*		*	*		*	*	*	6
60	Liow MHL	*		*	*	**	*	*	*	8
61	Sarda PK	*		*	*	*	*	*	*	7
62	Morris MJ	*		*	*		*	*	*	6
63	Van Jonberg en Jonberg en HPW	*		*	*	*	*	*	*	7
64	J. M. F.van Wagenb erg	*		*	*		*	*	*	6
65	Yadav B	*		*	*		*	*	*	6
66	Zicaro JP	*		*	*		*	*	*	6
67	Leadbet ter WB	*		*	*		*	*	*	6
68	Odumen ya M	*		*	*		*	*	*	6
69	Romagn oli S	*		*	*		*	*	*	6
70	Dejour D	*		*	*		*	*	*	6
71	Diane L. Dahm	*		*	*		*	*	*	6
72	Dahm DL	*		*	*	**	*	*	*	8
73	deDeug d CM	*	*	*	*	**	*	*	*	9
74	Halai M	*		*	*	*	*	*	*	7
75	kazarian	*		*	*		*	*	*	6
76	m de Winter, Rhijn Feith and Corné J M van Loon	*		*	*		*	*	*	6

All the data were carefully extracted from all eligible studies independently by the two reviewers (P.C. and M.R.). The data extracted from the selected studies included various variables: study title, authors, year of publication, study design, sample size, number of patients, number of males, number of females, number of patellofemoral prosthesis, prosthesis model types, age of patients, number of complications, types of complications, duration of follow‐up, rates of reoperation, type of reoperation and clinical outcomes. Any disagreement was resolved by discussion and consensus.

### Outcome measures

The primary outcome measure focused on radiological and functional outcomes and complications rates between inlay and onlay PFA for isolated PFOA. The secondary outcomes analysed included comparison of functional outcomes and postoperative complications of different implant types and models.

### Quality assessment

Two investigators (B.D. and M.C.) independently evaluated the quality of the full texts with consensus discussion in case of discrepancies. Quality assessment was performed with the Newcastle–Ottawa Quality Assessment Scale (Table 1).

### Risk of bias

We assessed risk of bias using the *Risk of Bias in Non‐randomized Studies of Interventions* (ROBINS‐I) framework, which evaluates seven key domains of potential bias. These domains are: (1) random sequence generation; (2) allocation concealment; (3) blinding of participants and personnel; (4) blinding of outcome assessment; (5) incomplete outcome data; (6) selective reporting; and (7) other potential sources of bias. Each domain was rated as ‘low’, “moderate” or “high” risk. In cases of discrepancy, two reviewers discussed their assessments and, if consensus was not reached, a third reviewer was consulted (Table 2).

**Table 2:** Risk of bias assessment of the studies using ROBINS‐I tool.

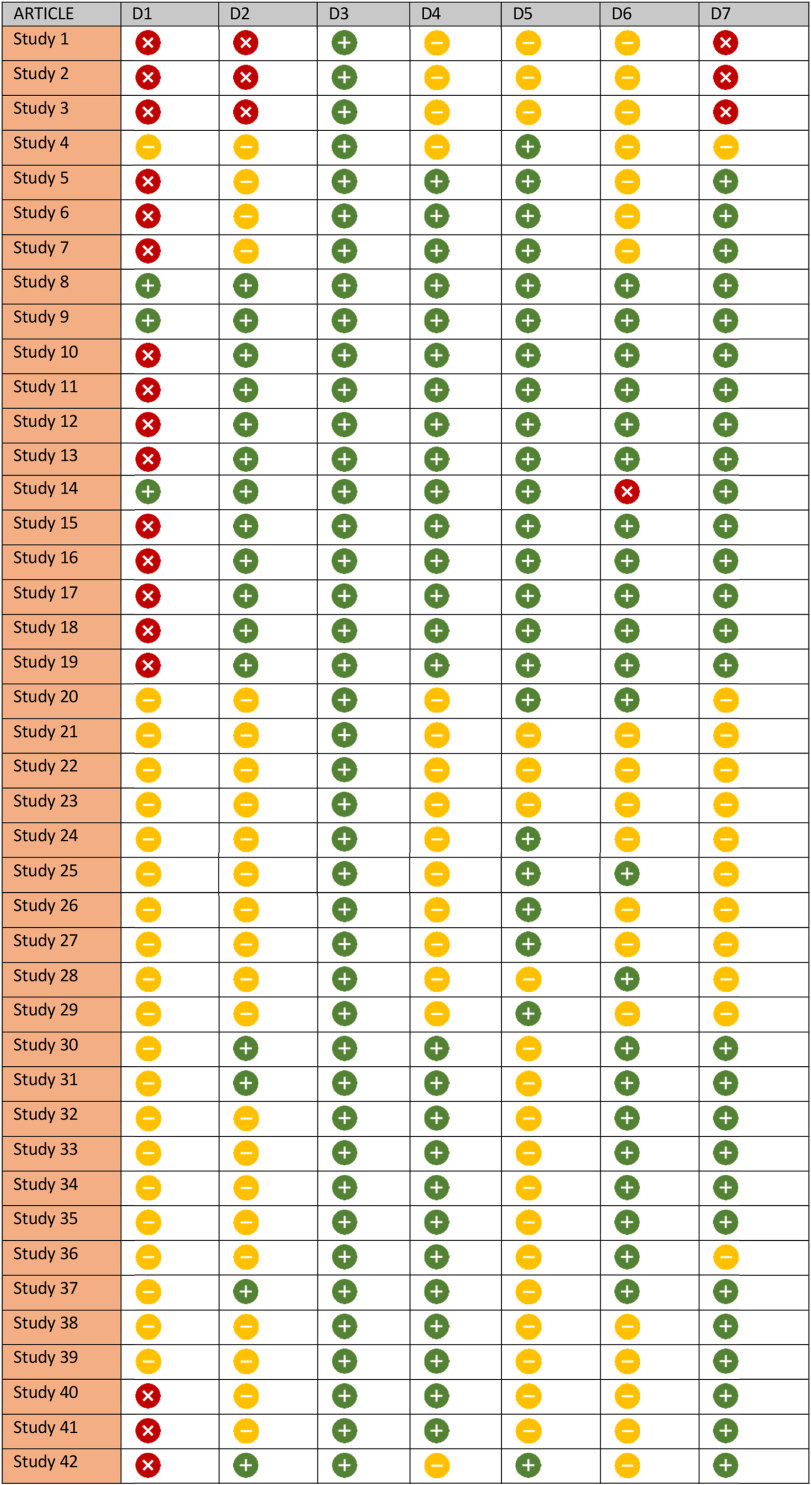
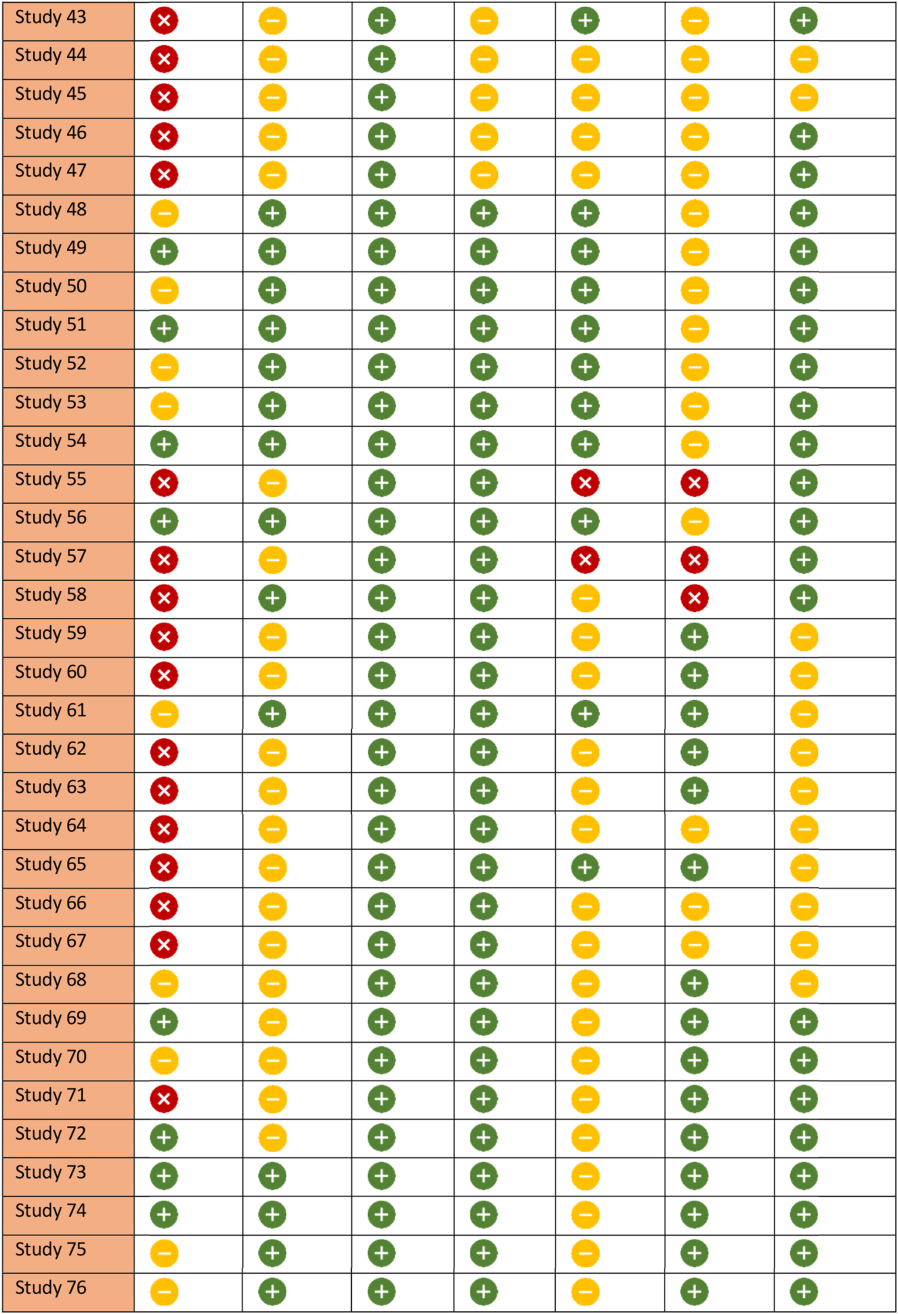

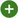
 Low risk of bias

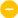
 Unclear risk of bias

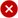
 High risk of bias

Domains:

D1: Random sequence generation (selection bias) D2: Allocation concealment (selection bias).

D3: Blinding of participants and personnel (performance bias) D4: Blinding of outcome assessment (detection bias).

D5: Incomplete outcome data (attrition bias) D6: Selective reporting (reporting bias).

D7: Other bias.

## STATISTICAL ANALYSIS

A meta‐analysis of proportions was performed using the metaprop package of STATA vers.18 (StataCorp LLC.) (Nyaga et al.) The prevalence data were transformed using the Freeman–Tukey double arcsine method to stabilize variance, particularly when proportions approach extreme values near 0% or 100%, which can distort meta‐analytic results.

This transformation enhances the reliability of pooled prevalence estimates by mitigating the undue influence of studies with extreme prevalence rates. Due to the expected heterogeneity, the metaprop function was used to combine proportions—defined as the number of events over the total number of observations—using a maximum likelihood model restricted to random effects (REML). To compare studies using the onlay versus inlay technique, aggregate incidences were calculated for each subgroup and indirect comparisons were made using the subgroup heterogeneity test. Forest plot diagrams were created for good/excellent results and postoperative complications according to the surgical technique used (inlay vs. onlay) and the type of prosthesis used.

## RESULTS

A total of 76 studies were included, encompassing 4484 patients (71.1% female; *n* = 3189). Among these, 2576 patients (57.5%) received an onlay‐type patellofemoral prosthesis, while 1908 patients (42.5%) received an inlay‐type implant. In total, 5084 patellofemoral prostheses were implanted, of which 2946 (57.95%) were onlay designs and 2138 (42.05%) were inlay designs.

The overall mean age of participants was 56.13 years. Breaking this down by prosthesis type, patients receiving onlay implants had a mean age of 57.31, whereas those receiving inlay implants had a mean age of 54.53. The mean follow‐up duration was 4.8 ± 3.2 years overall; specifically, the onlay subgroup had a mean follow‐up of 4.6 ± 2.6 years, and the inlay subgroup had a mean follow‐up of 5.1 ± 3.7 years.

The most commonly implanted onlay prostheses were the Avon (Stryker) design, with 1693 implants reported across 19 studies; the Zimmer Gender Solutions implant, with 537 implants across 8 studies; and the Journey (Smith & Nephew), with 327 implants across 5 studies. In the inlay category, the predominant designs were the HemiCAP Wave (Arthrosurface) with 598 implants in 12 studies, Richards (Smith & Nephew) with 526 implants in 7 studies, and Lubinus (Link) with 289 implants in 6 studies.

**Figure 2:**
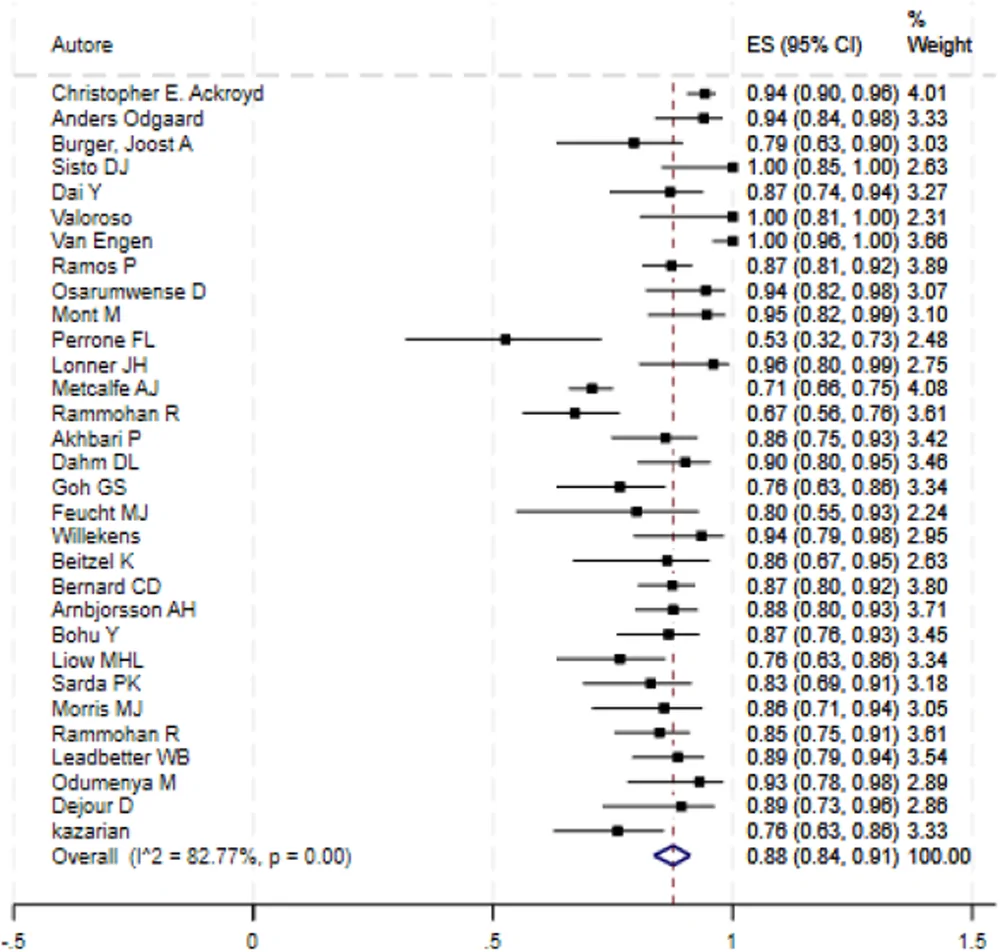
Forest plot for excellent/good clinical outcomes incidence in onlay femoropatellar prostheses.

**Figure 3:**
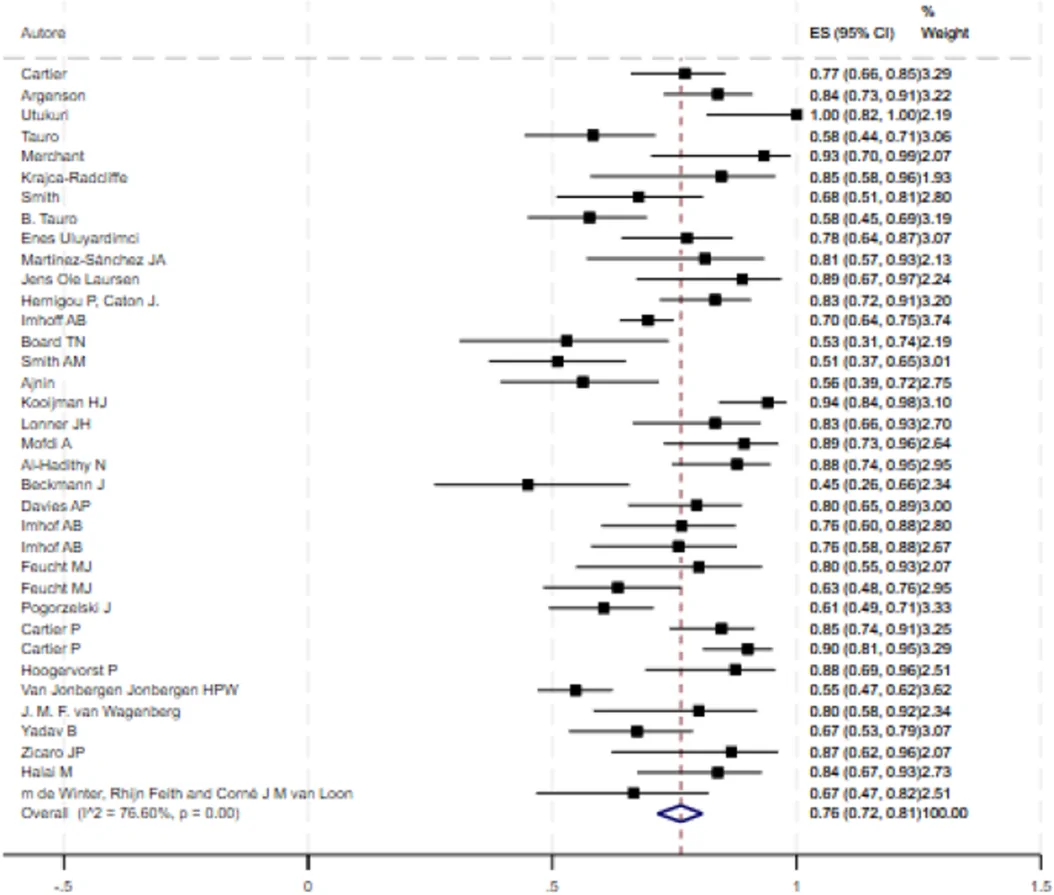
Forest plot for excellent/good clinical outcomes incidence in inlay femoropatellar prostheses.

A total of femoro‐patellar prostheses demonstrated an overall good to excellent clinical outcome rate of 82% (95% CI: 79–85). When stratified by prosthesis design, the onlay group showed a significantly higher rate of good/excellent outcomes at 88% (84–91%) compared to 76% (72–81%) in the inlay group (*p* < 0.001) (Figures 2 and 3). All onlay patellofemoral prosthesis models demonstrated a high incidence of good clinical outcomes (Tables 3 and 4). The highest rates were observed with the Avon (Stryker) prosthesis, reporting a 90% incidence of favorable outcomes (95% CI: 0.83–0.94), followed by the Gender Solutions (Zimmer) prosthesis at 87% (95% CI: 0.75–0.96). In contrast, clinical outcomes among inlay prostheses were more variable. While certain inlay models, such as the PFJ Vialla and Richards (Smith & Nephew), showed high rates of good clinical results, others—most notably the Lubinus (Link) prosthesis—were associated with considerably lower success rates, as detailed in Table 4.

**Table 3:** Pooled incidence for outcomes comparing inlay versus onlay technique.

	Total	Onlay technique	Inlay technique	*p* value
Excellent/good clinical outcomes	0.82 (0.79–0.85)	0.88 (0.84–0.91)	0.76 (0.72–0.81)	Random: Test for heterogeneity between sub groups: 14.311 0.001
	67 studies	31 studies	36 studies	
Postoperative complication*	0.12 (0.09–0.15)	0.07 (0.04–0.10)	0.19 (0.14–0.25)	17.781 0.001
	73 studies	37 studies	36 studies	
Patellar maltracking	0.02 (0.01–0.03)	0.01 (0.001–0.01)	0.04 (0.02–0.07)	14.451 0.001
	53	28	24	
Persistent postoperative pain	0.02 (0.01–0.04)	0.01 (0.001–0.02)	0.04 (0.01–0.11)	0.08
	37	13	24	
Infections	<0.001	<0.001	<0.001	0.19
	71	35	36	
Mechanical loosening	<0.001	<0.001	<0.001	0.5
	72	35	37	

**Table 4:** Excellent/good clinical outcomes for patellofemoral prosthesis models.

Prosthesis model	Incidence	IC 95%	N. studies
Richards S&N	0.80	0.67–0.91	7 inlay
Others	0.86	0.81–0.91	15 (8on e 7in)
Lubinus Link	0.62	0.52–0.71	6 inlay
Avon Stryker	0.90	0.83–0.94	13 onlay
HemiCAP Wave	0.72	0.66–0.78	11 inlay
Zimmer Gender Solutions	0.87	0.75–0.96	7 onlay
Vialla	0.80	0.69–0.90	5 inlay
Journey	0.79	0.68–0.89	5 onlay

**Figure 4:**
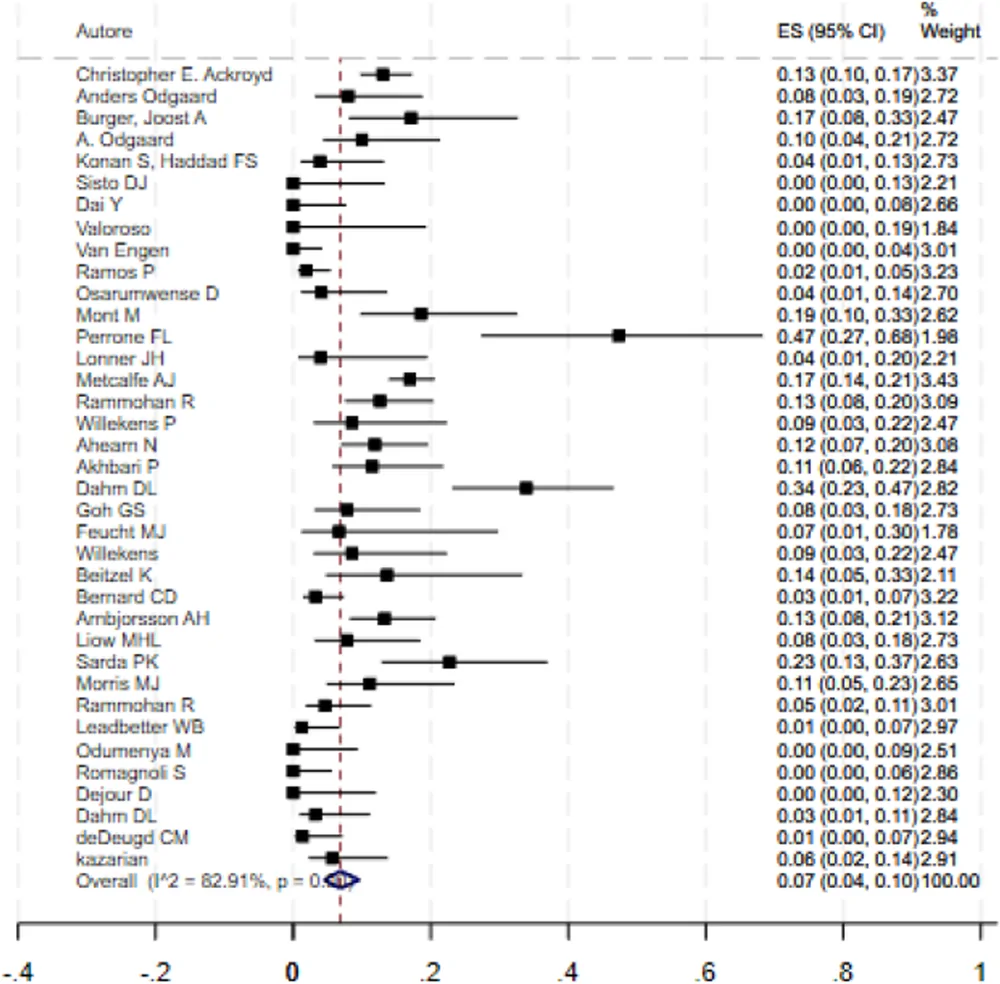
Forest plot for postoperative complications incidence in onlay femoropatellar prostheses.

**Figure 5:**
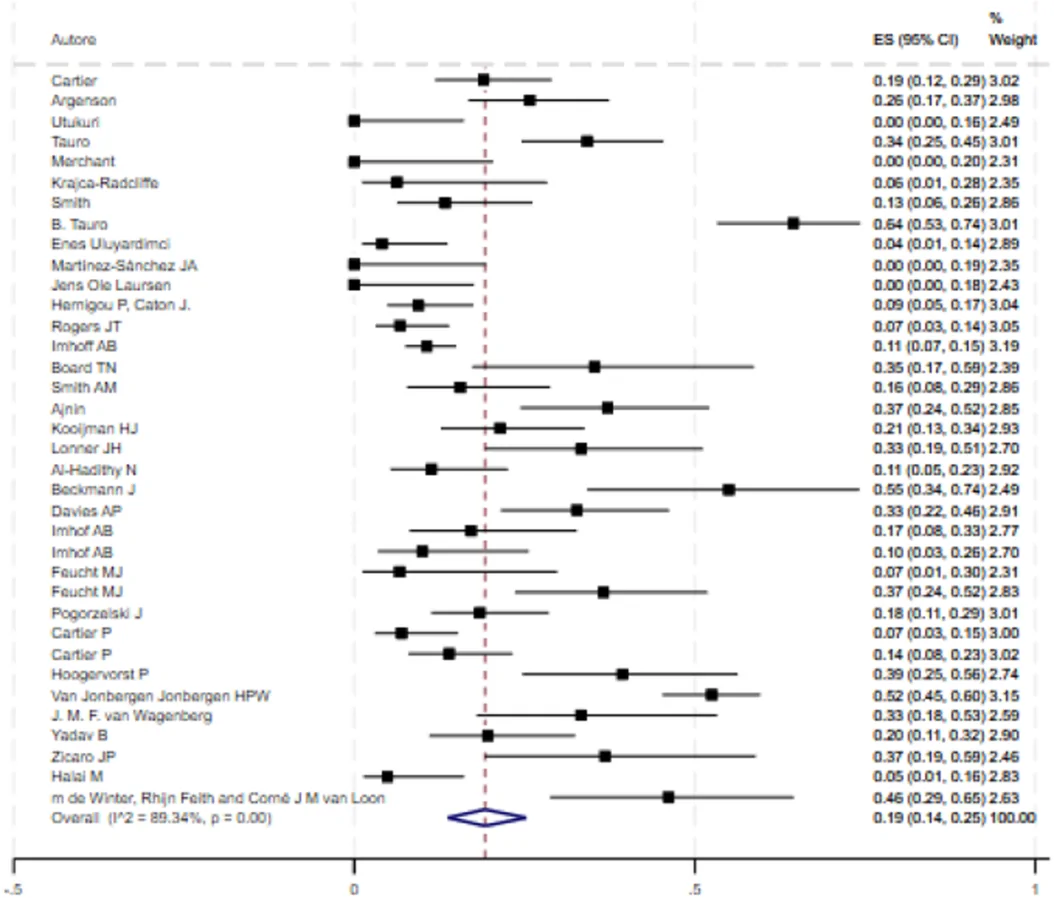
Forest plot for postoperative complications incidence in inlay femoropatellar prostheses.

The postoperative patellar maltracking rate was 2% overall (1%–3%), with 1% (0%–1%) in the onlay group and 4% (2%–7%) in the inlay group; this difference did not reach statistical significance (*p* < 0.001). Both infection and aseptic loosening rates were negligible at 0% for both groups (Table 3). The overall postoperative complication rate was 12% (0.9%–15%), with a significantly lower incidence in the onlay prostheses at 7% (4%–10%) versus 19% (14%–25%) in the inlay group (*p* < 0.001) (Table 3, Figures 4 and 5).

In the inlay group, the HemiCAP Wave exhibited the lowest complication rate at 15% (95% CI: 7%–23%), whereas the Richards (S&N) and Lubinus (Link) prostheses had higher complication rates of 27% (95% CI: 13%–43%) and 32% (95% CI: 16%–50%), respectively (Table 5).

**Table 5:** Postoperative complications for patellofemoral prosthesis models.

Prosthesis model	Incidence	IC 95%	N. studies
Richards S&N	0.27	0.13–0.43	7 in
Others	0.08	0.03–0.13	15 (7on e 8in)
Lubinus Link	0.32	0.16–0.50	6 in
Avon Stryker	0.08	0.05–0.13	17 onlay
HemiCAP Wave	0.15	0.07–0.23	11 inlay
Zimmer Gender Solutions	0.04	0.001–0.10	8 onlay
Vialla	0.20	0.07–0.37	4 inlay
Journey	0.09	0.06–0.13	5 onlay

Among onlay models, the Gender Solutions (Zimmer) prosthesis had the lowest complication rate at 4% (95% CI: 0%–10%), followed by the Avon (Stryker) at 8% (95% CI: 5%–13%) (Table 6).

**Table 6:** Revision surgery for patellofemoral prosthesis models.

Prosthesis model	Incidence	IC 95%	N. studies
Richards S&N	0.23	0.10–0.38	7inlay
Others	0.09	0.06–0.14	16 (8in e 8on)
Lubinus Link	0.34	0.13–0.60	6inlay
Avon Stryker	0.07	0.03–0.12	18 on
HemiCAP Wave	0.11	0.06–0.18	11 in
Zimmer Gender Solutions	0.02	0.001–0.03	8 on
Vialla	0.10	0.03–0.19	4in
Journey	0.08	0.03–0.14	5on

Revision rates were also significantly lower in onlay prostheses compared to inlay designs (*p* < 0.001). Within the onlay group, the Gender Solutions (Zimmer) prosthesis showed the lowest revision rate at 2% (95% CI: 0%–3%), followed by the Avon (Stryker) at 7% (95% CI: 3%–12%). In the inlay group, the FPJ Vialla had the lowest revision rate at 10% (95% CI: 3%–19%), while the Richards (S&N) and Lubinus (Link) prostheses exhibited higher revision rates of 23% (95% CI: 10%–38%) and 34% (95% CI: 6%–14%), respectively (Table 6).

Revision surgery with TKA occurred in 6% of cases overall (4%–8%), with rates of 4% (2%–7%) and 8% (6%–11%) in the onlay and inlay groups, respectively (*p* = 0.02). The rate of surgical lateral release was 1% across all cases (0%–2%), with no significant difference between onlay (0%–3%) and inlay (1%–3%) prostheses (Table 7).

**Table 7:** Pooled incidence for revision surgeries comparing inlay versus onlay technique.

	Total	Onlay technique	Inlay technique	*p* value
Overall revision surgeries	0.10 (0.07–0.14)	0.05 (0.03–0.09)	0.16 (0.11–0.23)	0.00
	75	36	39	
Revision surgery with total knee replacement	0.06 (0.4–0.08)	0.04 (0.02–0.07)	0.08 (0.06–0.11)	0.02
	70	33	37	
Revision surgery with lateral release	0.01 (0.001–0.02)	0.01 (0.001–0.03)	0.01 (0.01–0.03)	0.77
	46	22	24	

## DISCUSSION

Our comparative analysis of prosthetic models shows a clinical advantage of onlay patellofemoral prostheses over inlay designs. Onlay models such as the Avon (Stryker) and Gender Solutions (Zimmer) achieved the highest rates of favorable clinical outcomes and were also associated with lower postoperative complication and revision rates. These findings align with the presumed biomechanical benefits of onlay prostheses, including improved patellar tracking and more physiological joint kinematics.

Our findings suggest that onlay patellofemoral prostheses may offer clinical advantages over inlay designs, although the observational nature of most included studies, study heterogeneity, and medium‐term follow‐up should be considered when interpreting these results.

The overall rate of good to excellent outcomes was significantly higher in the onlay group, particularly for the Avon (Stryker) and Gender Solutions (Zimmer) prostheses, which achieved success rates of 90% and 87%, respectively. These results are consistent with existing literature. For instance, Ackroyd et al. [2] reported a 3.6% failure rate in 306 Avon prostheses, and Anders Odgaard et al. [46] reported 94% excellent or good clinical outcomes in 50 Avon implants. Further support for the Avon prosthesis comes from Akhbari et al. [5], Willekens et al. [58], Sarda et al. [22, 56], Leadbetter et al. [38] and Odumenya et al. [47], all of whom reported favorable clinical outcomes. Similarly, Van Engen et al. [56] reported a 0% failure rate in 87 Gender Solutions implants, and Dai et al. [16] observed 87% excellent or good results in a cohort of 46 implants. Positive outcomes with the Gender Solutions prosthesis have also been confirmed by Kazarian et al. [34]

Postoperative complications were significantly less frequent in the onlay group, further supporting the reliability of these implants. Notably, the Gender Solutions (Zimmer) model demonstrated the lowest complication and revision rates. In contrast, inlay prostheses—particularly the Lubinus (Link)—were associated with substantially higher risks. Tauro et al. [54] reported less than 50% excellent or good clinical outcomes in 76 Lubinus implants, while Board et al. [9] observed that 4 out of 17 Lubinus inlay prostheses required conversion to TKA at a mean follow‐up of 1.6 years.

The patellar maltracking rate, although numerically higher in the inlay group, did not reach statistical significance, suggesting that surgical technique and individual anatomical variability may also contribute to this outcome. Importantly, the incidence of both infection and aseptic loosening was negligible across all models, reflecting advances in surgical protocols and implant design. The significantly lower need for revision surgeries and conversion to TKA in the onlay group further supports their clinical durability.

The marked variability among inlay prostheses suggests that implant design plays a critical role in determining clinical performance. For example, while the Vialla inlay prosthesis showed mixed results—with Ajnin [4] reporting 7 reoperations (including 5 revisions to TKA) out of 43 implants at a mean follow‐up of 5.4 years. Al‐Hadithy et al. [6] reported a 97% survival rate at 3 years in a series of 49 Vialla patellofemoral prostheses. Similarly, outcomes with the Richards (Smith & Nephew) inlay prosthesis were variable. Hoogervorst et al. [30] and Van Jonbergen et al. [57] reported high failure and revision rates, while Cartier [14] reported approximately 90% excellent or good results in 79 implants at a 10‐year follow‐up.

In contrast, the HemiCAP PF Wave inlay prosthesis demonstrated consistently poor performance. Beckmann et al. [7] reported a success rate of less than 50% in 20 implants, with 11 requiring revision to an onlay‐type prosthesis. Similar findings were reported by Pogorzelski et al. [48], who also observed low success rates and high revision rates. Although most evidence trends toward better medium‐term outcomes with onlay designs, some matched‐pair data (e.g., HemiCAP Wave vs. Journey PFJ) found no difference in clinical scores but observed less tibiofemoral OA progression in the inlay group [21]. This suggests that in certain anatomical or alignment settings, inlay prostheses may perform comparably or even have specific advantages.

The variability observed among inlay prostheses is unlikely to be incidental and may be explained by multiple factors. First, implant design has evolved considerably: earlier models such as the Lubinus had limited anatomical design and poorer control of patellar tracking, while more recent inlay designs like the Richards or Vialla incorporated modifications that yielded more favorable clinical results. Second, inlay implants are often used in younger and more active patients, which may contribute to higher revision rates regardless of prosthesis design. Third, inlay implantation is technically demanding, as it requires precise mapping of the native trochlea; errors in depth or positioning can negatively influence outcomes. Finally, many inlay studies are limited by small sample sizes and heterogeneous outcome reporting, which may exaggerate inconsistencies. Taken together, these elements provide a more robust explanation for the heterogeneous performance of inlay implants.

These findings underscore the importance of prosthesis‐specific design features and highlight the necessity of careful implant selection based on patient‐specific anatomical and functional demands. Notably, the consistently low rates of infection and aseptic loosening across all prosthetic types may reflect advancements in surgical technique, patient selection, and perioperative management.

## LIMITATIONS

The results of this study should be interpreted with caution in light of several limitations. First, the comparison between surgical techniques (onlay vs. inlay) was conducted through indirect subgroup analyses, as individual patient data and head‐to‐head randomized studies were not available. Second, the meta‐analyses showed, in some cases, wide confidence intervals due to the presence of rare outcomes and the substantial heterogeneity across populations, surgical techniques, and prosthetic types. This review is limited by the predominance of non‐randomized, retrospective studies and by the moderate risk of bias in several domains. The mean follow‐up of 4.8 years is relatively short for arthroplasty survival analysis and the heterogeneity of outcome measures complicates direct comparisons. However, the pooled estimates covers a broad range of implant models, allowing a comprehensive comparison of device‐specific outcomes. These factors mean that conclusions should be interpreted as indicative trends rather than definitive evidence.

## CONCLUSIONS

These findings suggest that onlay PFA appears to offer clinical advantages over inlay designs in the treatment of isolated PFOA at medium‐term follow‐up, with trends toward superior functional outcomes, lower complication and revision rates. Onlay patellofemoral prostheses are associated with superior clinical outcomes, lower complication rates, and reduced need for revision surgery compared to inlay designs. Among the evaluated models, the Avon (Stryker) and Gender Solutions (Zimmer) prostheses demonstrated the most favorable results, while the Lubinus (Link) inlay prosthesis was associated with the poorest outcomes. These findings support the preferential use of onlay prostheses in PFA and emphasize the critical role of implant selection in surgical success. However, further prospective, longer‐term and randomized studies are warranted to validate these results and to better understand the factors influencing implant‐specific performance.

## AUTHOR CONTRIBUTIONS

The author(s) read and approved the final manuscript.

## CONFLICT OF INTEREST STATEMENT

The authors declare no conflicts of interest.

## ETHICS STATEMENT

The authors have nothing to report.

## Data Availability

Data available on request from the authors.
